# Prosecution for Mal-Practice

**Published:** 1846-08

**Authors:** 


ARTICLE XVI.

### PROSECUTION FOR MAL-PRACTICE. ARBITRATION.

John Beals,<br>vs.<br>Nathan M. Thomas. } This was an action for damages in the Circuit Court of Kalamazoo, Mich., which had once been brought to trial before that court, the Hon. Epaphroditus Ransom, Chief Justice of the State of Michigan, presiding, when the jury returned a verdict of $300 damages. A new trial had been granted, but at the June term it was taken out of court and referred to Dr. Zina Pitcher, of Detroit, Michigan, and Dr. Daniel Brainard, of Chicago, Ill., as arbitrators, with power to choose a third person in case of disagreement.

The arbitration was held at the Court House in Kalamazoo, June 12, 1846. The most material facts of the case were stated by the witnesses as follows: Daphne Beals, daughter of John Beals, in August, 1841, being at the time about 11 years of age, was see-sawing on a board placed across a fence, and fell, injuring her left fore-arm and elbow. Doctor Thomas was called, and by the aid of an assistant made some extension and counter extension, and other manipulations, reduced and dressed it, saying it was a fracture of one of the bones. The dressings consisted of a roller, applied from the hand to the elbow, splints with compresses on the anterior and posterior sides, secured with a roller, and the fore-arm placed in a sling across the breast, according to some witnesses, at less than a right angle with the arm. (There was some discrepancy in the testimony on this point.)

These dressings were renewed every eight days, and the forearm flexed and extended during eight weeks when they were discontinued. The arm, according to the testimony of the girl and family, being in the same state as at present.

On examining the arm it presents the following appearances: 1st. The head of the radius can be felt with perfect distinctness, and the prominence produced by its presence, seen in front of its natural position and upon the edge of the ulna. 2d. Every movement of the fore-arm and hand, flexion and extension, pronation and supination, can be completely performed. 3d. In flexion and pronation, the head of the radius returns to its natural position, but glides from it in extension and supination. 4th. Pressure upon the head of the radius also returns it, and the displacement is immediately reproduced upon removing the pressure.

Those familiar with the signs of dislocation of the head of the radius forwards, will immediately perceive that it is a case of that accident not attended by the usual symtoms. These are laid down by Sir Astley Cooper, thus: "The fore-arm is slightly bent, but cannot be brought to a right angle with the upper, nor can it be completely extended.

"When it is suddenly bent, the head of the radius strikes against the fore part of the os humeri, and produces so sudden a stop to its motion as at once to convince the surgeon that one bone strikes against the other, the hand is placed in a prone position, but neither its pronation nor supination can be completely performed.

"If the thumb be carried into the fore and upper part of the elbow joint, the head of the radius can be felt there, and if rotation of the hand be attempted, the bone will be perceived to roll; this last circumstance, and the sudden stop to the bending of the arm, are the best diagnostic marks of the injury."

Of these all but one, it will be seen, were absent in this case. The head of the bone could be felt, but when the fore arm was flexed, it did not arrest it, but striking against the os humeri feebly, it glided into its place.

From the manner of the accident the absence of all signs of fracture, taken in connexion with its present state, the arbitrators were of opinion that it was a case of dislocation, which, if reduced, had not been retained so, and they were obliged therefore to enquire what damage the plaintiff had sustained, and how far the defendant was responsible for it.

In relation to the first point we have already said that all the movements of the arm were complete, and it may be added that the muscular development of that arm (a good index of its power,) was nearly as great as in the other. It was not a case of loss of a member from unskilfulness, still deformity, and a certain degree of inconvenience, resulted, and this, it is probable, might have been prevented, had the nature of the accident been recognized at the time.

But the accident did not present the ordinary signs, and if those only laid down by the best authorities are to be relied upon, it could not be a dislocation forward. These signs, in some cases are not present. This was a case of this kind, presenting therefore unusual difficulties, and liable to be mistaken by persons possessed of *ordinary* surgical skill. Dr. Thomas, therefore, could not be held responsible for the damage resulting from the injury, if he exercised care and diligence and ordinary skill. Care and diligence were no doubt used by the defendant in this case. He appears to be a physician of accredited skill, highly honorable character, and unpretending. According to the principles of law, as laid down by the highest authorities recently, no physician is liable for damages, if he exercises the utmost of his skill. On this head we quote the following decisions from the St. Louis Medical and Surgical Journal, for May, 1846.

"'*Norfolk Lent Assizes*, April 5, 1845; before Baron Parke. Gibbs *vs.* Tunnaley. This was an action to recover damages for negligence and want of professional skill as a medical man, in the treatment of an injury of the arm. The Court held, "That, if, in the discharge of his duty, the physician *applied his professional skill and knowledge to the best of his ability*, then, however unfortunate the termination of the case, he was not to be visited with an action to mulct him in damages."'

"Same Assizes, Queen *vs.* Raymond Gaches, before Baron Parke; trial for manslaughter, in dragging away the womb instead of the placenta. 'The learned Judge in summing up said, that he perfectly agreed with Lord Ellenborough, that if a medical man was to be punished for the death of a patient, whenever he used *the most skill he was possessed of*, such would be fraught with much danger. The prisoner, no doubt, at first, thought it was a simple tumor he was bringing away—that was a proof that it was an error in judgment. They might perhaps say he had a right to know; but how could a man learn without some practice? and if a man happened to make an error in judgment, was that to be considered gross ignorance, and imputed to criminal neglect? Doubtless, *if the man had known better, he would not have done so.*'

"These cases may be found at full length in the American reprint of the London Lancet, (for which they were specially reported) for July, 1845. In the same journal, for March, 1845, pp. 267-8, we find the following additional case:

"'*Midland Circuit.* Forcible Inversion of the Womb; trial for Manslaughter, before Mr. Justice Patterson, who held in his charge to the jury, "That, *if a person having skill and knowledge made an accidental mistake in treatment, through which the patient died, he was not guilty of manslaughter;* it would be fearful if he were.'"

Called upon to decide a case involving the rights of medical men generally, it was impossible not to remember that the same laws which render us liable to damages for ignorance, impose penalties for pursuing the study of anatomy, and that the State of Michigan, unlike the most liberal and enlightened governments of modern times, affords no sufficient protection for this branch of Science. There was but one point on which the arbitrators could not fully approve of the conduct of the defendant; it was in not advising the patient to seek the best surgical advice within her reach, in a case in which his own experience must of necessity have been limited.

On this ground, while shielding him from damages, they thought it best so to draw up their award as to avoid expressing in every respect, full approbation of the course he pursued.

### AWARD.

The undersigned, to whom was submitted the matter in difference between John Beals and Nathan M. Thomas, having carefully examined the injured limb of Daphne Beals, and heard the testimony adduced in relation to the injury and its treatment, and the arguments of council in the cause, respectfully report to the Honorable Chief Justice of the State of Michigan, presiding over the Circuit Court of Kalamazoo County.

That they agree in the opinion, that the said injury was a dislocation of the upper end of the radius forwards not detected at the time of its occurrence, and that the defendant is therefore liable to the imputation of mal-practice, from defective anatomical knowledge.

Taking into consideration that this dislocation is one of rare occurrence, and has in several cases been found to be incapable of reduction, and that it was not in this instance attended by the usual distinguishing signs, and was obscured by considerable swelling:

Considering farther, that the study of anatomy essential to the proper treatment of such cases, is by the laws of the State of Michigan a penitentiary offence; together with the fact, that the limb is still highly useful and may, in our opinion, be essentially improved by judicious treatment, we award that the defendant in this case, is not justly liable to any damages, and (if it belongs to the board to decide this question,) we further award that each party shall pay his own costs.

ZINA PITCHER.
DANIEL BRAINARD.

Kalamazoo, June 13, 1846.

